# An online survey of Irish general practitioner experience of and attitude toward managing problem alcohol use

**DOI:** 10.1186/s12875-018-0889-0

**Published:** 2018-12-18

**Authors:** Claire Collins, Pearse Finegan, Margaret O’Riordan

**Affiliations:** Irish College of General Practitioners, 4-5 Lincoln Place, Dublin 2 County Tipperary, Ireland

**Keywords:** Alcohol, General practice, Screening, Intervention, Management

## Abstract

**Background:**

In the general practice setting screening, brief intervention and counselling have been shown to be effective in the reduction of problem alcohol use. This study aimed to explore Irish general practitioners’ (GPs) current practice of and attitudes towards the management of problem alcohol use.

**Methods:**

An online survey was emailed, with one email reminder, to 1750 general/family practitioners who were members of the Irish College of General Practitioners (ICGP) and for whom an active email address was available. Overall, 476 completed questionnaires were received representing a 27.2% response rate.

**Results:**

Two-thirds of the respondents reported that they have managed patients for problem alcohol use and related issues in the past year. The majority, 96%, of respondents indicated that they initiate conversations around alcohol even when the patient does not do so. Almost two thirds of GPs stated that they use structured brief intervention when talking to patients about their alcohol intake and circa 85% reported that they provide some form of counselling in relation to reducing alcohol consumption. While more than two out of three GPs felt prepared when counselling patients in relation to alcohol consumption, almost half considered they are ineffective in helping patients to reduce alcohol consumption. One third of GPs advised that they did not have access to an addiction counsellor.

**Conclusions:**

GPs in this survey reported widespread experience of screening and intervention, however, many still felt ineffective. In order to maximise the potential impact of GPs, a clearer understanding is required of what interventions are effective in different scenarios. Furthermore, GPs are only part of the solution in terms of addressing alcohol consumption. The services available in the broader health care system and Government alcohol related policy needs to further support GPs and patients.

**Electronic supplementary material:**

The online version of this article (10.1186/s12875-018-0889-0) contains supplementary material, which is available to authorized users.

## Background

Alcohol use disorders are as common and as costly as both depression and coronary heart disease [1]. Alcohol consumption is now linked to more than 60 medical conditions [2]. Alcohol problems hinder efforts towards the assessment and treatment of these conditions [2] while also increasing risky behaviours [3].

Irish residents rank among the highest consumers of alcohol and of binge drinkers in Europe showing increasing rates while other countries are showing reductions [1, 4, 5]. Alcohol use is embedded within the cultural fabric of the nation, receiving a level of accommodation extended to no other drug [6, 7].

The harm experienced both by the individual and by those in their personal or social vicinity as a result of problem alcohol use in Ireland was previously highlighted [8].Taking just two examples, alcohol was found to be a contributory factor in 36.5% of all fatal road accidents, while it was also described as a main trigger in 34% of domestic violence cases [9].

The high level of alcohol consumption in Ireland and the concomitant harm it produces is imposing substantial tangible costs upon the Irish Exchequer with the main burden being placed upon the health care and criminal justice systems. Based upon an assessment of data from 2007, the estimated overall annual cost of problem alcohol use is €3.7 billion, accounting for 1.9% of GDP for that year. Such tangible costs are above the European average for the percentage of GDP spent on alcohol misuse (1.3%) and are at the higher end of this cost range within Europe (0.9 to 2.4%) [10].

Due to increased population contact and as part of a health promotion role, primary care settings are in a unique position for alcohol identification screening and intervention [11–14].

The identification and assessment of those who are at risk of, or already experiencing alcohol-related difficulties is of fundamental importance. It is accepted that clinical interviewing has outperformed the chemical or biological marker forms of assessment [15]. Upon consideration of a number of reviews of brief screening tools, the Alcohol Use Disorders Identification Test (AUDIT) and CAGE questionnaire (the name ‘CAGE’ is an acronym of the four questions contained in this tool) are the most widely used and validated screening tools in primary care settings [16, 17].

Brief interventions within primary care settings, typically following screening, are valuable in the management of individuals with alcohol-related problems [14, 18]. While, it is acknowledged that there may be confusion over what constitutes ‘brief intervention’, it is generally accepted to be more than providing feedback on risk level and the provision of a leaflet only [19].

The evidence indicates that brief intervention in primary care settings can lower levels of alcohol consumption [20–24] with the advantages of low associated costs [25, 26] and limited added time for practitioners [27–30]. It has also been demonstrated that behavioural counselling can reduce alcohol consumption [31–33]. As many as 20% of patients in Ireland have been shown to have unhealthy drinking patterns [6]. The evidence suggests that brief intervention can help to reduce drinking levels across all patient groups [3].

The ICGP has conducted much work in this area with guidelines, courses, e-learning modules and research having been embarked upon since 2000 [34–41]. This study aimed to explore Irish GPs current practice of and attitudes towards the management of problem alcohol use.

## Methods

The ICGP is the professional body for GPs in Ireland representing over 90% of GPs in Ireland. An online survey via SurveyMonkey was emailed, with one email reminder, to 1750 ICGP members for whom email addresses were available and up to date.

The questionnaire (shown in Additional file 1) was designed by the project advisory group and included (with permission) questions from a similar study -the Optimizing Delivery of Health Care Interventions (ODHIN) project [42] in order to permit international comparison. The ODHIN project involved nine European countries and aimed to improve the delivery of health care interventions. It focused on the implementation of identification and brief intervention programmes related to alcohol consumption. Analysis was undertaken using descriptive statistics with SPSS (Version 21).

## Results

Overall, 476 completed questionnaires were received giving a 27.2% response rate. Respondent demographics and comparison to the ICGP member population are shown in Table 1. Over three-quarters (77.7%) were engaged in seven or more general practice clinical sessions per week and 62.5% saw more than 100 patients weekly.

**Table 1:** Respondent Profile Demographics

	Respondents %	Population^a^%
Gender		
Male	47.3	47.2
Female	52.7	52.8
Age Group		
< 30	1.3	1.6
30–39	31.8	30.0
40–49	24.4	27.0
50–59	27.7	28.4
60–69	14.9	13.0

^a^Irish College of General Practitioner Member Statistics 2016

**Fig. 1:**
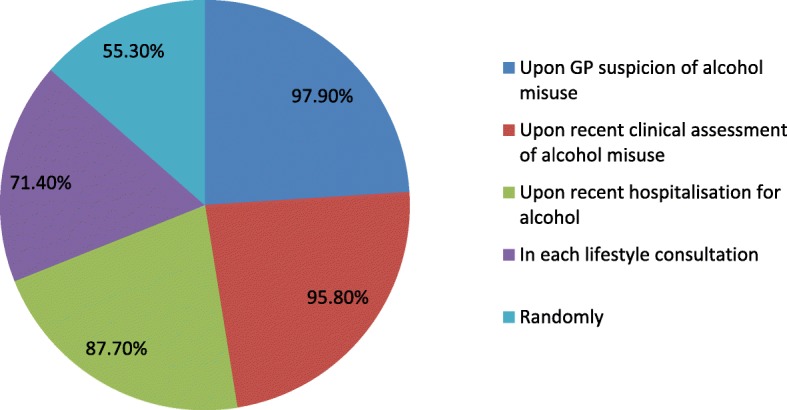
Scenarios in which the screening tool/discussion is used (% of GPs reporting use in each scenario)

Overall 42.7% reported that they screened for alcohol misuse among their patients with 42.4% of these using a specific screening tool, with that employed most often being the CAGE questionnaire (88.9%). Almost three-quarters of the respondents used the screening tool (71.4%) in all lifestyle consultations and 87.8% to 97.9% in situations of specific concern/relevance (Fig. 1). Over half (55.3%) used the tool randomly in consultations even when lifestyle or specific concerns were not present.

Of those who responded, 30.5% stated that in the scenario where the patient does not mention alcohol, they ask about alcohol consumption all or most of the time, 66.4% do so some of the time while the remaining 4.1% rarely or never do so.

Almost all GPs (96.3%) felt they have the right to ask patients about their drinking when necessary and 79.2% felt that their patients consider they have a right to do so. Overall, 86.3% agreed that they can appropriately advise their patients about drinking and its effects and 67.7% agreed that they know enough about the causes of problem drinking to carry out their role in this area (Table 2).

**Table 2:** GPs agreement/disagreement with statements about working with people who are dependent on alcohol or have a severe problem with alcohol

	Agree	Neither agree nor disagree	Disagree
I feel I know enough about the causes of drinking problems to carry out my role when working with drinkers	67.7% (*n* = 226)	19.8% (*n* = 66)	12.5% (*n* = 41)
I feel I can appropriately advise my patients about drinking and its effects	88.2% (*n* = 294)	8.1% (*n* = 27)	3.7% (*n* = 12)
I feel I have the right to ask patients questions about their drinking when necessary	96.4% (*n* = 321)	3.6% (*n* = 12)	-(*n* = 0)
I feel that my patients believe I have the right to ask them questions about drinking when necessary	79.3% (*n* = 264)	14.4% (*n* = 48)	6.3% (*n* = 21)

Two-thirds of GPs who responded reported that they have managed patients for hazardous drinking and alcohol dependency and related alcohol problems in the last year. Almost two-thirds (62.8%) reported using structured brief intervention when talking to patients about their alcohol intake and 84.7% reported providing some form of counselling in relation to reducing alcohol consumption. When treating a patient with an alcohol problem, 15.5% of GPs recommended total abstinence, 16.7% a reduction in intake of alcohol and 67.8% reported that they did not have a pre-determined approach but adapted this to suit the patient. Over half (52.7%) of GPs were applying weekly alcohol consumption limit guidelines (17 and 11 standard drinks per week for males and females respectively [37]) on which to base their advice to patients. A further 28.1% used a cut off which was lower than that indicated in the guidelines while 19.2% used a cut-off higher than recommended weekly limits.

Overall, 63.2% of GPs who responded had direct access to addiction counsellors while 32.6% had direct access to a residential addiction service. Four-fifths (79.6%) of the respondents referred a patient in the past year to the psychiatry service (including addiction counsellors).

**Fig. 2:**
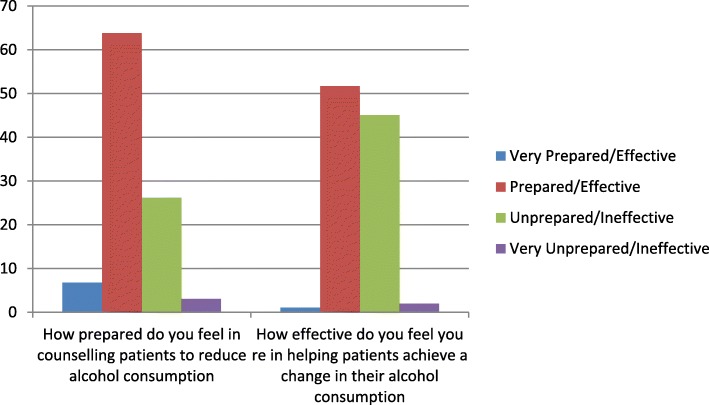
GPs perceived preparedness and effectiveness with regard to counselling patients in relation to reducing alcohol consumption

While more than two out of three GPs felt prepared when counselling patients in relation to alcohol consumption, almost half (49.7%) considered they were ineffective in helping patients to reduce alcohol consumption (Fig. 2). Respondents to this survey were very positive with regard to the potential effectiveness of GPs in reducing patients’ alcohol consumption with 32.2% considering they would be very effective and 58.5% effective if given adequate information and training. The remaining 9.3% thought they would be ineffective.

## Discussion

### Main findings

Almost all GPs who responded to this survey initiated conversations around alcohol even when the patient did not do so. The majority of GPs reported that they have managed patients for hazardous drinking and alcohol dependency and related alcohol problems in the past year. When treating a patient with an alcohol problem, the majority of respondents indicated their approach to abstinence was patient dependent. The latter likely reflects the GPs’ knowledge of their patients, taking account of the patient’s medical and family history and support networks. While more than two out of three GPs felt prepared when counselling patients in relation to alcohol consumption, almost half considered they were ineffective in helping patients to reduce alcohol consumption. This survey highlighted the disparity of access to support services for GPs and their patients with alcohol problems with some who did not have access to an addiction counsellor.

### Limitations of the study

This response rate, which may be considered as low, is in fact typical of surveys of physicians internationally [43, 44]. Low response rates raise concerns about bias and it could be the case that such a survey results in under or over estimates. However, the concern of bias is somewhat negated by the distribution across all demographic descriptors and as the demographic distribution was representative of all ICGP members [45].

### Interpretation of findings in the context of existing evidence

In Ireland, more than half (54.3%) of those aged 18–75 years who consume alcohol are classified as harmful drinkers [46]. GPs in Ireland at an individual level have significant experience in dealing with alcohol related problems, given the extent to which their patients experience ongoing difficulties and harm from alcohol consumption.

In the general practice setting, screening [16, 47–50], brief intervention [14, 20, 21, 24], and counselling [31, 32] for early problems related to alcohol have been shown to be effective in the reduction of harmful and hazardous drinking. The results of our survey showed that the majority of GPs in this survey reported using structured brief intervention when talking to patients about their alcohol intake and most provided some form of counselling in relation to reducing alcohol consumption.

Similar to the findings from the ODHIN study, conducted in nine other European countries, GPs in our survey called for better training and infrastructure [42].

Over half of the respondents felt they were ineffective in terms of helping patients to reduce alcohol consumption. Many factors may contribute to this and not least the fact that we need to better understand how, why, when and what brief interventions work [51–53]. Furthermore, our respondents may be accurate in terms of patient acceptance of being asked about their alcohol consumption, as found elsewhere [54], however, the impact of intervention may be limited if patients do not view their drinking as problematic [55] and it may not lead to increased use of alcohol-related care [51].

### Implications for research and practice

Other recent research has shown that the documentation of alcohol consumption status and of the interventions undertaken is poor in Irish general practice [56] and this may have implications for effectiveness in terms of follow-up as shown in other scenarios [57, 58]. We did not look at patient records in this study but further research on the level of brief intervention, its recording and the resultant patient outcomes in the Irish setting would be valuable. However, screening and intervention by health professionals is only one aspect in terms of addressing the impact of alcohol consumption on our health, community and associated alcohol related costs [51, 53, 59]. A full review of policies is required and necessitates collaboration between Government departments and agencies and across the full health system [51, 53, 59].

## Conclusions

The impact of interventions in the general practice setting may be limited in a country such as Ireland with high levels of alcohol consumption, given that acceptance and potentially a lack of recognition among patients that their consumption level is problematic. GPs in this survey reported widespread experience of screening and intervention, however, many still felt ineffective. In order to maximise the potential impact of GPs, a clearer understanding is required of what interventions are effective in different scenarios. Furthermore, GPs are only part of the solution in terms of addressing alcohol consumption. The services available in the broader health care system and Government alcohol related policy needs to further support GPs and patients.

## Additional file

Additional file 1: Study questionnaire. Questionnaire_GP experience of managing problem alcohol use. (PDF 275 kb)

## Abbreviations

AUDIT: Alcohol Use Disorders Identification Test
CAGE: The CAGE questionnaire, the name of which is an acronym of its four questions, is a widely used screening test for problem drinking and potential alcohol problems
GP: General practitioner
HSE: Health Service Executive
ICGP: Irish College of General Practitioners
ODHIN: Optimizing Delivery of Health Care Interventions
OECD: The organisation for economic co-operation and development
WHO: World Health Organisation
