# Supplementary material for: An online survey of Irish general practitioner experience of and attitude toward managing problem alcohol use
Source: BMC Fam Pract. 2018 Dec 18;19:200. doi: 10.1186/s12875-018-0889-0 (PMC6297939; doi:10.1186/s12875-018-0889-0)
Supplement: Supplementary file 1 — Study questionnaire. Questionnaire_GP experience of managing problem alcohol use. (PDF 275 kb) [file 12875_2018_889_MOESM1_ESM.pdf]

Survey of GP experience of managing problem alcohol use

GP experience of dealing with alcohol mis-use

The high level of alcohol consumption in Ireland and the concomitant harm it produces is an issue Irish GPs know about and deal with frequently both in terms of the individual and his/her family. However, little has been formally documented about GP experience of providing alcohol related services, the challenges posed, and the need for training and support in this regard.
In order to provide a picture of this and to obtain your opinion of how future interventions might be planned, the ICGP are conducting an online survey. You have been randomly selected to receive this survey, which will take <15 minutes to complete.
You cannot be identified in any way from your completed questionnaire and confidentiality is assured. The responses received will be aggregated during analysis and no individual can be identified.

Please complete each section by ticking the relevant box or write your answers in the space provided. Please select one answer unless otherwise indicated.

1. Section A: DEMOGRAPHICS

Your Gender:

◯ Male ◯ Female

2. Your Age Group:

◯ <30 ◯ 30-39 ◯ 40-49 ◯ 50-59 ◯ 60-69 ◯ 70+

3. Number of years in General Practice:

◯ <5 ◯ 5-<15 ◯ 15<30 ◯ 30+

4. Please indicate your primary practice location:

◯ City ◯ Town ◯ Village

5. Please indicate which best describes your primary practice:

◯ Group ◯ Single-handed

6. How many clinical sessions in general practice do you work each week?

◯ 0 ◯ 1-2 ◯ 3-4 ◯ 5-6 ◯ 7-8 ◯ 9+

7. How many general practice patients would you see in an average week?

- ◯ 0-50
- ◯ 51-100
- ◯ 101-150
- ◯ More than 150

Section B: Alcohol/Alcohol Related Problems Training and Experience

8. If the patient doesn't ask you about alcohol, do you ask about it?

○ All the time ○ Most of the time ○ Some of the time ○ Rarely or never

9. Do you screen for alcohol abuse among your patients?

○ Yes ○ No

10. Do you use a specific alcohol abuse screening or identification tool?

- ◯ Yes
- ◯ No

11. If Yes, which tools do you use, please tick ALL that apply

- [ ] Alcohol Use Disorder Identification Test (AUDIT)
- [ ] FAST Test
- [ ] AUDIT-C Test
- [ ] CAGE Questionnaire

12. As you do not use a screening tool, please describe the main strategy you employ in order to instigate a discussion in relation to a patient’s use/mis-use of alcohol:

13. Is this screening tool/strategy for discussion of possible alcohol mis-use utilised in the following scenario?

	Yes	No
Upon GP suspicion of alcohol misuse	◯	◯
Upon recent clinical assessment of alcohol misuse symptoms	◯	◯
Completed upon recent hospitalisation for alcohol abuse related illness/circumstance	◯	◯
Randomly	◯	◯
In each lifestyle consultation	◯	◯

14. Are you satisfied with the effectiveness of the procedures employed in relation to identifying alcohol mis-use in your practice?

◯ Yes ◯ No

(If NO please outline the challenges you have experienced in this regard.

15. Do you use structured brief intervention when talking to patients about their alcohol intake?

◯ Yes ◯ No

16. Do you provide any form of counselling in relation to reducing alcohol consumption?

◯ Yes ◯ No

17. Doctors vary in their counselling skills and training. How prepared do you feel when counselling patients in relation to reducing alcohol consumption?

◯ Very Prepared ◯ Prepared ◯ Unprepared ◯ Very Unprepared

18. How effective do you feel you are in helping patients achieve change in relation to reducing alcohol consumption?

◯ Very Effective ◯ Effective ◯ Ineffective ◯ Very Ineffective

19. When treating a patient with an alcohol problem do you seek total absence or a reduction in intake of alcohol?

◯ Total Absence ◯ Reduction in Intake ◯ Varies, Patient Dependent

20. Do you use standard drinks, units of alcohol or grams of alcohol when talking to your patients?

◯ Standard Drinks ◯ Units of Alcohol ◯ Grams of Alcohol

21. For a healthy adult man, what would you consider the upper limit for alcohol consumption per week before you would advise him to cut down? Please enter your answer in standard drinks or units of alcohol or grams of alcohol.

Standard Drinks per week ____________________

Units of alcohol per week ____________________

Grams of alcohol per week ____________________

22. For a healthy adult woman, who is not pregnant, what would you consider the upper limit for alcohol consumption per week before you would advise her to cut down? Please enter your answer in standard drinks or units of alcohol or grams of alcohol.

Standard Drinks per week ____________________

Units of alcohol per week ____________________

Grams of alcohol per week ____________________

23. In the last year, how many times have you taken or requested a blood test (eg MCV, GGT) because of concern about alcohol consumption?

◯ Never

◯ 1 – 2 times

◯ 3 – 5 times

◯ 6 – 12 times

◯ More than 12 times

24. In the last year, how many patients have you managed for (a) hazardous drinking or alcohol-related problems or (b) alcohol dependency and related alcohol problems

hazardous drinking or alcohol-related problems

alcohol dependency and related alcohol problems

25. Please indicate in the table below, how many of your alcohol dependent patients have started on each type of detox programme in the last year, how many of these completed the detox programme and how many are still alcohol free.

	Number started on detox	Number completed detox	Number still alcohol free
Voluntary detox programme e.g. non Government agencies			
Community detox programme – HSE administered			
Hospital based detox – public			
Hospital based detox – private (e.g. St Pats/John of Gods)			
GP Detox programme- initiated and monitored by you/GP			

26. Thinking of your most recent patient who commenced a GP initiated detox programme, what care givers were actively involved in their acute (first 7 days) detox programme? Please tick ALL that apply.

- GP
- Practice Nurse
- Support Worker/Key Worker
- Community Addiction Services
- Outpatient Hospital Services
- Pharmacist
- Community Psychiatry services
- Addition Counsellor
- HSE alcohol detox Nurse Practitioner

Other (please specify)

27. Do you have direct access to addiction counsellors?

- ◯ Yes ◯ No

28. Do you have direct access to a residential addiction service?

- ◯ Yes ◯ No

29. How often in the past year have you made a referral to the psychiatry service (including addiction counsellors) for a patient with alcohol addiction?

- ◯ None
- ◯ 1 – 6 times
- ◯ 7 – 12 times
- ◯ 13 or more times

Section C: Views and Opinions

Section C: Views and Opinions

Thank you for persevering with this survey - you are nearing completion.

30. Please indicate how much you agree or disagree with each of the following statements about working with people who are dependent on alcohol or have a severe problem with alcohol.

	Strongly Agree	Quite Strongly Agree	Agree	Neither Agree nor Disagree	Disagree	Quite Strongly Disagree	Strongly Disagree
I feel I know enough about the causes of drinking problems to carry out my role when working with drinkers.	◯	◯	◯	◯	◯	◯	◯
I feel I can appropriately advise my patients about drinking and its effects.	◯	◯	◯	◯	◯	◯	◯
I feel I have the right to ask patients questions about their drinking when necessary.	◯	◯	◯	◯	◯	◯	◯
I feel that my patients believe I have the right to ask them questions about drinking when necessary.	◯	◯	◯	◯	◯	◯	◯

31. If a GP were to initiate a patient on a patient on an alcohol detox programme, what do you consider should be available to support this (Please tick ALL that apply).

- ☐ Irish Guidelines on Community Detox
- ☐ Education and Training for GPs
- ☐ Education for Patients
- ☐ Protected time
- ☐ Timely access to services

Other (please specify)

32. In terms of supporting GPs who choose to initiate a patient on a community alcohol detox programme, what other care givers do you feel should be involved in the acute (first 7 days) phase of the programme [please number these from 1 to 8 in order of priority].

	Rating Scale
Practice Nurse	☐
Support Worker/Key Worker	☐
Community Addiction Services	☐
Outpatient Hospital Services	☐
Pharmacist	☐
Community Psychiatry services	☐
Addition Counsellor	☐
HSE alcohol detox Nurse Practitioner	☐

33. In general, given adequate information and training, how effective do you feel general practitioners could be in helping patients change behaviour in relation to reducing alcohol consumption?

○ Very Effective ○ Effective ○ Ineffective ○ Very Ineffective
